# NMR Structure of the Myristylated Feline Immunodeficiency Virus Matrix Protein

**DOI:** 10.3390/v7052210

**Published:** 2015-04-30

**Authors:** Lola A. Brown, Cassiah Cox, Janae Baptiste, Holly Summers, Ryan Button, Kennedy Bahlow, Vaughn Spurrier, Jenna Kyser, Benjamin G. Luttge, Lillian Kuo, Eric O. Freed, Michael F. Summers

**Affiliations:** 1 Howard Hughes Medical Institute, University of Maryland Baltimore County, 1000 Hilltop Circle, Baltimore, MD 21250, USA; E-Mails: lola.brown@yale.edu (L.A.B.); cassiah.cox@nih.gov (C.C.); janaeb@umbc.edu (J.B.); hsummers@umbc.edu (H.S.); ryan.button@umaryland.edu (R.B.); kennedy.bahlow@temple.edu (K.B.); vspurrier@uchicago.edu (V.S.); jmk216@lehigh.edu (J.K.); 2 Virus-Cell Interaction Section, HIV Drug Resistance Program, National Cancer Institute at Frederick, Frederick, MD 21702-1201, USA; E-Mails: bxl244@case.edu (B.G.L.); kuols@mail.nih.gov (L.K.)

**Keywords:** Feline immunodeficiency virus (FIV), nuclear magnetic resonance (NMR), protein structure, retrovirus assembly, membrane targeting, phosphatidylinositol-4,5-bisphosphate [PI(4,5)-P_2_, PIP_2_]

## Abstract

Membrane targeting by the Gag proteins of the human immunodeficiency viruses (HIV types-1 and -2) is mediated by Gag’s N-terminally myristylated matrix (MA) domain and is dependent on cellular phosphatidylinositol-4,5-bisphosphate [PI(4,5)P_2_]. To determine if other lentiviruses employ a similar membrane targeting mechanism, we initiated studies of the feline immunodeficiency virus (FIV), a widespread feline pathogen with potential utility for development of human therapeutics. Bacterial co-translational myristylation was facilitated by mutation of two amino acids near the amino-terminus of the protein (Q5A/G6S; myrMA^Q5A/G6S^). These substitutions did not affect virus assembly or release from transfected cells. NMR studies revealed that the myristyl group is buried within a hydrophobic pocket in a manner that is structurally similar to that observed for the myristylated HIV-1 protein. Comparisons with a recent crystal structure of the unmyristylated FIV protein [myr(-)MA] indicate that only small changes in helix orientation are required to accommodate the sequestered myr group. Depletion of PI(4,5)P_2_ from the plasma membrane of FIV-infected CRFK cells inhibited production of FIV particles, indicating that, like HIV, FIV hijacks the PI(4,5)P_2_ cellular signaling system to direct intracellular Gag trafficking during virus assembly.

## 1. Introduction

Assembly of human immunodeficiency virus (HIV) particles occurs by a complex, multistep mechanism that includes temporally and spatially dependent interactions with several cellular host factors at or near the plasma membrane (PM) [1,2,3,4,5,6]. Several thousand copies of Gag self-associate to form a single virus particle, which is enveloped by a lipid bilayer derived from the host cell [5]. The viral envelope of HIV-1 is enriched in phosphatidylinositol-4,5-bisphosphate [PI(4,5)P_2_], cholesterol, and lipid constituents associated with lipid raft-like microdomains [7,8,9,10,11,12,13], indicating that virus assembly occurs in rafts. Membrane binding is dependent on a basic surface patch that is conserved among known strains of HIV-1, HIV-2, and SIV, as well as an N-terminal myristyl group that is added co-translationally during ribosomal protein synthesis.

Targeting of Gag to PM assembly sites is mediated by PI(4,5)P_2_ [14,15,16,17,18,19], a member of a family of differentially phosphorylated phosphatidylinositides that serve as membrane markers for cellular proteins [20,21,22,23]. PI(4,5)P_2_ is normally enriched in the inner leaflet of the PM [21], and depletion of PI(4,5)P_2_ inhibits virus assembly and leads to accumulation of Gag at the membranes of late endosomes and multivesicular bodies (MVBs) [14]. In addition, induction of PI(4,5)P_2_-enriched endosomes can retarget Gag to endosomes and MVBs and induce intravesicle budding, and substitution of HIV-1 MA by the membrane-binding N terminus of Fyn kinase reduces the sensitivity of virus assembly to PI(4,5)P_2_ manipulation. These studies collectively indicate that PI(4,5)P_2_-dependent membrane selection is mediated by the MA domain of Gag [14]. NMR studies of HIV-1 and HIV-2 myrMA indicate that PI(4,5)P_2_ binds to a surface cleft common to both proteins, and that binding triggers myristate exposure [17,24]. *In vitro* binding assays with other lipid constituents containing truncated acyl chains have led to proposals that phosphatidylserine, phosphatidylethanolamine, and phosphatidylcholine may also bind HIV-1 myrMA in extended lipid conformations, and thereby help target Gag to PM rafts [25].

Other retroviruses also appear to utilize a PI(4,5)P_2_-dependent membrane targeting mechanism. Moloney murine leukemia virus (MoMuLV), an evolutionarily distant gammaretrovirus that encodes an N-terminally myristylated Gag protein, is dependent on PI(4,5)P_2_ for membrane targeting and virus production [26]. The Gag protein of equine infectious anemia virus (EIAV) also binds PI(4,5)P_2_ and other phosphoinositides, but this protein is not myristylated and appears to target internal membranes prior to viral release [19,27]. Studies with the betaretrovirus Mason-Pfizer monkey virus (M-PMV) revealed a functional dependence of PI(4,5)P_2_ for PM targeting, despite the fact that M-PMV Gag proteins assemble in periplasmic regions of the cell before associating with the PM [28].

We have initiated efforts to understand the structural and functional roles of MA in feline immunodeficiency virus (FIV) assembly. FIV is a lentivirus that infects about 8% of the feline population in the United States and is gaining interest as a model for therapeutic and vaccine development [29,30]. Felines provide an alternative to HIV animal models [31,32,33,34]—they have an immune system similar to that of humans, are easily housed and maintained, and cost about ten times less than simians [35]. Additionally, studies on FIV have been shown to be translatable to HIV [36,37,38]. Felines were the initial animal model used in FDA-approved HIV-1 integrase and reverse transcriptase inhibitor studies [38]. Additionally, the first *in vivo* studies demonstrating that nucleoside analogs are an effective HIV-1 drug target were performed in felines [39,40]. This work led to the development of Tenofovir, a drug frequently used in HIV-1 HAART therapy [38]. In addition to similarities in the clinical response between HIV and FIV, the genomic organization and viral life cycle of FIV are similar to those of HIV. Mutational studies indicate that, as observed for HIV-1, membrane binding is dependent on a basic surface patch and N-terminal myristylation [41,42].

Recently, the structure of the unmyristylated form of FIV MA [(myr(-)MA] was determined by X-ray crystallography [43]. The protein adopts a globular structure similar to that observed for the HIV and SIV myr(-)MA proteins. The structure also contains features that were not observed in the HIV and SIV MA structures, including relatively long peptide stretches that were ordered but did not adopt canonical α-helical or β-sheet type conformations. In addition, dynamic light scattering and protein cross-linking suggested that FIV MA forms a dimer in mildly acidic solutions and at high protein concentration (400 μM, pH 6.0) [43]. In contrast, HIV-1 myr(-)MA was observed to be monomeric in solution under similar conditions, even at high concentrations [44,45], and both HIV-1 and SIV myr(-) MA proteins were found to crystallize as trimers [46,47,48]. Recent cryo-electron microscopic studies indicate that the MA domains of HIV-1 Gag do not form an ordered oligomeric array when bound to the inner membranes of immature particles [49]. Although the FIV myr(-)MA structure is of high quality and the studies provided important new insights, several questions emerged, particularly regarding the location of the acyl chain and potential structural alterations that might occur in the myristylated form of the protein [43]. It is also important to know if FIV assembly is dependent on PI(4,5)P_2_. To address these questions, we have conducted solution-state NMR studies with a recombinant form of FIV MA, engineered to facilitate bacterial co-translational myristylation, as well as virus assembly/release studies with MA-mutant and wild-type viruses.

## 2. Materials and Methods

### 2.1. Plasmid Cloning

The FIV MA gene was PCR amplified from the Petaluma strain, clone 34TF10 (accession code NC_001482) kindly provided by J. Elder (Scripps Research Institute, La Jolla, CA) through the National Institutes of Health (NIH) AIDS Research and Reference Reagent Program) and cloned into the pET 11a co-expression vector followed by a nucleotide sequence for a 6× histidine tag. The co-expression vector also contained the yeast N-terminal myristyltransferase (yNMT) gene. To allow for myristylation of FIV MA using yNMT, Q5A/G6S mutations were introduced into the FIV MA gene using QuikChange Kit according to manufacturer’s instructions (Stratagene, La Jolla, CA, USA). Plasmids were sequenced through the University of Maryland Sequencing Facility (College Park, MD, USA) or Genewiz, Inc. (South Plainfield, NJ, USA).

### 2.2. Protein Expression

BL21 DE3(RIL) *E. coli* cells (Agilent Technologies, Tewksbury, MA, USA) transformed with the FIV MA/yNMT plasmid were grown in 4 L of Luria Broth medium supplemented with either 10 mg/L unlabeled myristic acid or uniformly labeled ^13^C myristic acid (Sigma Aldrich, St. Louis, MO, USA) at 37 °C until the OD_600_ reached between 0.6–0.7. The cells were centrifuged and washed with 1 × M9 salt before transferring them to 1 L of M9 minimal medium containing ^15^N-NH_4_Cl and/or ^13^C glucose (Cambridge Isotope, Tewksbury, MA, USA) as the sole carbon and nitrogen source. The cells shook for 1 h before induction with 1mM isopropyl-D-thiogalactoside (Sigma Aldrich). Cells were grown at 30 °C for 12–14 h and lysed using a microfluidizer (Microfluidics, Westwood, MA, USA). The proteins were purified by cobalt affinity chromatography (Clontech, Mountain View, CA, USA) and cation exchange SP column chromatography (GE Life Science, Piscataway, NJ, USA). Molecular weights and efficiency of myristylation were confirmed by electrospray ionization mass spectrometry (David King, UC Berkeley, or Molecular Characterization and Analysis Complex, UMBC. Representative result: expected 15532.4, actual 15532.6 ± 0.3, ≥95% myristylation). Samples for all NMR experiments were prepared in 50 mM sodium phosphate, 150 mM NaCl, 10 mM DTT, pH 7.0.

### 2.3. NMR Spectroscopy

NMR data were collected with Bruker DMX (600 MHz ^1^H, 800 MHz ^1^H) spectrometers equipped with cryoprobes, processed with NMRPIPE [50] and analyzed with NMRViewJ [51,52]. A combination of two-, three- and four-dimensional NOESY data were obtained for combinations of natural abundance, ^15^N- and ^15^N-,^13^C-labeled protein samples (20 °C) [53,54,55,56]. Protein backbone signals were assigned using standard triple resonance methods [57]. Intermolecular ^1^H-^1^H NOEs between ^15^N-,^13^C-labeled protein and unlabeled myristate were assigned using 2D ^1^H-^1^H NOESY [58] and 2D ^1^H-^13^C HMQC [59] datasets.

### 2.4. Structure Calculations

Structures were calculated in torsion angle space with CYANA [60]. Upper interproton distance bounds of 2.7, 3.3 and 5.0 Å (with appropriate corrections for pseudoatoms) were employed for NOE cross peaks of strong, medium, and weak intensity respectively, which were qualitatively determined following intensity normalization of the different NOE data sets. We were unable to obtain residual dipolar couplings due to precipitation that occurred when myrMA was introduced to gels. Structure figures were generated with PyMOL [61]. The electrostatic surface potential was generated using the Adaptive Poisson Boltszmann Solver (APBS) [62].

### 2.5. FIV Release Assays

HEK 293T cells (ATCC) were transfected with pFP93 (FIV Gag expression vector) or Crandall-Reese Feline Kidney (CRFK) cells (obtained from ATCC) were co-transfected with pFIV-Orf2rep (FIV-Petaluma proviral clone) and plasmids expressing human Arf6 in either its native or constitutively active form or human 5-phosphatase-IV (5ptaseIV) in its native or catalytically inactive form using Lipofectamine LTX and PLUS reagent (Invitrogen, Life Technologies, Grand Island, NY, USA), at a ratio of 9:1 (FIV:Arf6/5ptaseIV). The 5ptaseIV expression plasmid, pcDNA4TO/Myc5ptaseIV, was a kind gift from P. Majerus (Washington University School of Medicine, St. Louis, MO, USA). The Δ1 5ptaseIV mutant lacking the phosphatase signature domain has been described [14]. Plasmid expressing HA-tagged Arf6/Q67L, pXS/Arf6Q67L-HA, was a generous gift from J. Donaldson (National Heart, Lung, and Blood Institute, NIH). pFIV-Orf2rep is a derivative of pFIV-34TF10 (kind gift of J. Elder) that was modified by site-directed mutagenesis to restore encoding for full-length Orf2/Orf-A. The pFP93 vector (kind gift of E. Poeschla) expresses FIV Gag with a CMV promoter for studies in human cells. Methods for metabolic labeling and immunoprecipitation of FIV proteins were performed as described [33]. Transfected cells were metabolically radiolabeled with [^35^S] Met/Cys for 6 h. Cell and viral lysates were prepared and immunoprecipitated with mouse anti-FIV p24gag (clone PAK3-2C1). Gag proteins were quantified by phosphorimager analysis. Virus release efficiency was calculated as the amount of virion-associated Gag as a fraction of total (cell plus virion) Gag, normalized to samples containing FIV plasmid alone. Reverse transcriptase (RT) assays were performed as described previously [33].

## 3. Results

### 3.1. Construct Design

Attempts to prepare myristylated FIV MA using the dual expression vector developed for the HIV-1 myrMA [63] resulted in efficient production of myr(-)FIV MA protein and the yeast N-myristyltransferase (yNMT) enzyme but did not produce the desired myristylated FIV MA protein. This co-expression plasmid was previously shown to function more efficiently in *E. coli* than the human NMT [63]. Unfortunately, yNMT does not appear to be able to efficiently recognize the N-terminus of the FIV MA protein. The most common myristyl signal is M-G-X-X-X-S/T (M = methionine, G = glycine, S = serine, T = threonine, X = variable amino acid) [64], whereas the N-terminus of FIV MA contains a M-G-N-G-Q-G (N = asparagine; Q = glutamine) sequence. Mutations to alter the 6^th^ amino acid of FIV MA to a threonine resulted in a low level of myristylation when co-expressed with yNMT. Optimal myristylation was achieved with the double mutant, Q5A/G6S (myrMA^Q5A/G6S^).

### 3.2. N-Terminal Mutations do not Affect Virus Assembly or Release

**Figure 1:**
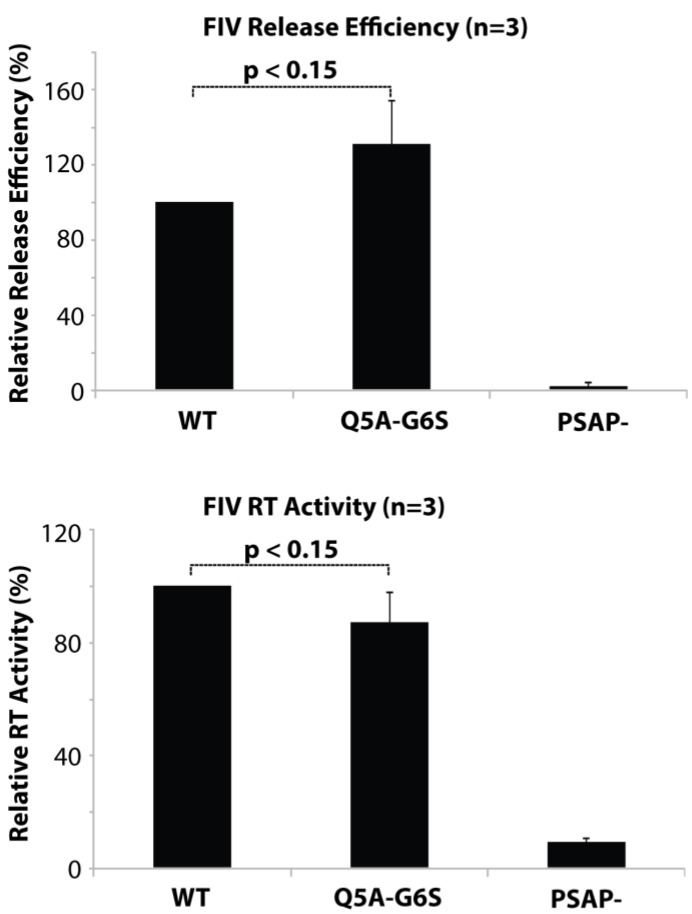
Mutation of the feline immunodeficiency virus (FIV) Gag myristylation signal does not significantly affect virus assembly or release from transfected 293T cells. Top panel, cells were metabolically radiolabeled with [^35^S] Met/Cys and FIV proteins were immunoprecipitated from cell and viral lysates with anti-FIV p24gag (see Materials and Methods). Virus release efficiency was calculated as the proportion of virion-associated FIV capsid compared with total (cell + virion) FIV Gag. Lower panel, virus release was determined by measuring the reverse transcriptase (RT) activity in the transfected cell medium. Error bars indicate standard error of the mean (SEM) from at least 3 independent experiments. Differences between WT and the Q5A/G6S mutant were determined to not be statistically significant by the paired Student’s *t*-test. PSAP- is an FIV late domain mutant that displays a severe defect in virus release [33] and thus serves as a negative control.

To determine if the mutations made to optimize myristylation of FIV MA by yNMT affect MA function, assembly and release assays were conducted (Figure 1). Viral release assays, performed on wild-type FIV and Q5A/G6S mutated viruses and assayed by ^35^S-Met/Cys radioactivity of viral lysates, revealed no significant differences in virus particle production (*p* < 0.15). Reverse transcriptase (RT) assays were also performed and showed no significant difference between wild-type and Q5A/G6S mutated viruses (*p* < 0.15). As a control, the budding-deficient late-domain mutant PSAP- [33] was included in the analysis. Together, these data indicate that Q5A/G6S mutations did not significantly affect FIV particle assembly or release.

### 3.3. NMR Signal Assignments

**Figure 2:**
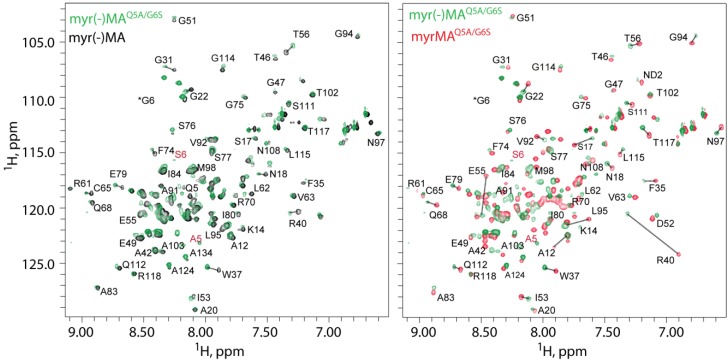
^1^H-^15^N HSQC of FIV myr(-)MA^Q5A/G6S^ (green) overlaid with wild-type FIV myr(-)MA (black, on left) and FIV myrMA^Q5A/G6S^ (red, on right). Residues marked in red indicate mutated residues (A5, S6). Asterisk denotes peak present in lower levels.

Both the unmyristylated and myristylated forms of FIV MA^Q5A/G6S^, and the unmyristylated form of wild-type FIV MA, were co-expressed in *E. coli* and purified by column chromatography. Purity and myristylation efficiency were verified by mass spectrometry. Two dimensional (2D) [^1^H–^15^N] HSQC spectra obtained for all three proteins were very similar, except for signals associated with residues Ser 17, Glu 32, Arg 40, Leu 60, Ser 101 and the stretch of residues near the N-terminus (G4–R7) (Figure 2). For all proteins, the ^1^H and ^15^N NMR signals were insensitive to concentration over the range of 50 µM to ~1 mM. These findings contrast with those obtained previously for HIV-1 myrMA, in which a subset of signals were shown to shift progressively toward the frequencies observed for myr(-)MA upon increasing the protein concentration. The changes observed for the HIV-1 protein were attributed to a concentration-dependent shift in a monomer-trimer equilibrium that occurs with concomitant exposure of the myristyl group [63]. The absence of concentration-dependent shifts for FIV myrMA^Q5A/G6S^ over the concentration range of 50–400 μM indicates that the protein exists in a unique conformation under these conditions.

**Figure 3:**
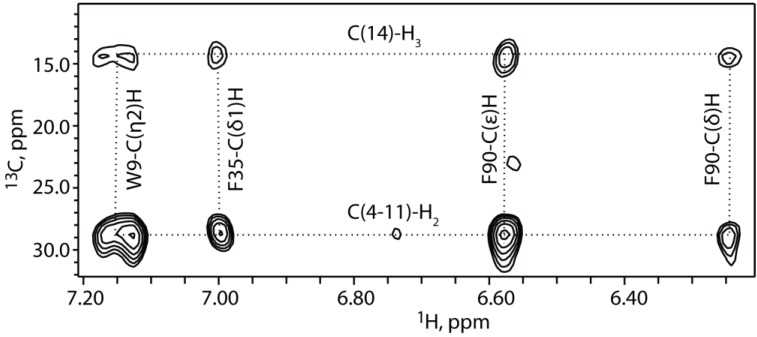
2D ^1^H-^13^C HMQC-NOESY data obtained for FIV myrMA^Q5A/G6S^ prepared using ^13^C-myristic acid, showing intermolecular NOEs between protons on FIV MA and ^13^C labeled myristyl group (myristate is uniformly ^13^C labeled, FIV MA at natural carbon abundance).

2D ^1^H-^1^H NOESY, 3D ^15^N edited NOE, and 2D ^1^H-^13^C HMQC NOE data obtained for FIV myrMA^Q5A/G6S^ samples prepared using uniformly labeled ^13^C-myristic acid exhibited NOEs between protons of the myristyl group and the side chains of Trp 9, Ile 53, Glu 55, Phe 90, Leu 60 and Ala 91. Representative portions of the 2D ^1^H-^13^C HMQC NOE data collected for myrMA^Q5A/G6S^ containing amino acids at natural isotopic abundance, but with uniformly ^13^C-enriched myristic acid, are shown in Figure 3. These data indicate that the myristyl group is buried within the core of the protein and makes contacts with the side chains of Trp 9, Ile 53, and Glu 55. In addition, strong NOE cross-peaks were observed between the terminal methyl group (myr-C^14^H_3_; ~0.65 ppm) and the side chains of Phe 90, Leu 60 and Ala 91, indicating a close packing of the myristyl group against these hydrophobic residues. In all spectra obtained, no intra-protein NOEs were detected that would be indicative of a significantly different myristate-dependent protein conformation, and the relatively small spectral differences observed for residues Ser 17, Glu 32, Arg 40, Leu 60, Ser 101 of the myr(-)MA^Q5A/G6S^ and myrMA^Q5A/G6S^ proteins reflect minor local adjustment to allow insertion of the myristyl group. NMR signal assignments have been deposited in the BMRB (http://www.bmrb.wisc.edu; accession number 25573).

### 3.4. Structure Determination

**Figure 4:**
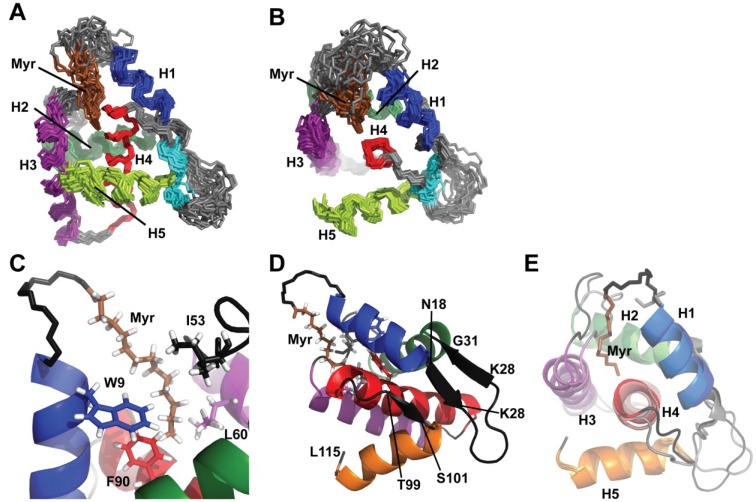
(**A**, **B**) Views of the NMR ensemble (20 structures, generated by superposition of backbone atoms of refined residues) showing the relative orientation of helices and position of the myristyl group; (**C**, **D**) Representative NMR structure showing (**C**) the packing of the myristyl group with the side chains of buried hydrophobic residues; and (**D**) relative position and orientation of the three-strand pseudo-β-sheet; (**E**) Comparison of a representative myrMA^Q5A/G6S^ NMR structure with the X-ray crystal structure of myr(-)MA.

A total of 835 interproton distance restraints, including 258 intraresidue, 275 sequential (|j-1| = 1), and 302 medium/long range restraints (|*j*-*i*| > 2) were derived from the NOE data. In addition, 302 hydrogen bonding restraints identified on the basis of NOE cross peak patterns and reduced H^N^ chemical exchange rates were used for structure calculations (Table 1). A set of 20 structures with total target functions (sum of the square of the distance violations) of 0.05 Å^2^ or less and maximum individual violations of 0.10 Å or less were generated for the intact protein using random initial atom coordinates. Considering the experimentally restrained residues (3–123, excluding glycines), 93.5% have backbone torsion angles that map to the most favored regions of the Ramachandran map, 5.7% are in additional allowed regions, and only 0.8% are in disallowed regions [65]. Statistical information (Table 1) and overlay of the best fit superpositions of the backbone atoms (Figure 4) demonstrate that the calculations afforded structures with low residual distance violations and good internal convergence. The atomic coordinates have been deposited in the Protein Data Bank (www.pdb.org; accession number 2N1R).

**Table 1:** Structure statistics for FIV MA^Q5A/G6S^.

**NMR-Derived Restraints ^1^**	
Interproton restraints	835
Intraresidue	258
Sequential (|i-j| = 1)	275
Medium/long range (|i-j| > 1)	302
Protein-myristate	8
^1^H-^1^H distance restraints	302
Total restraints	1137
Average restraints per residue	9
**Residual Restraint Violations**	
CYANA target function, Å^2^	0.04 ± 0.007
Maximum violations	
Upper limits, Å^2^	0.0053 ± 0.09
Lower limits, Å^2^	0.0057 ± 0.07
Van der Waals, Å^2^	0.7 ± 0.08
**Structure Convergence**	
Pairwise rms deviations^2^	
Backbone heavy atoms, Å^2^	0.96 ± 0.24
All heavy atoms, Å^2^	1.79 ± 0.39
**Ramachandran Analyses ^2^**	
Most favored regions, %	93.5
Additional allowed regions, %	5.7
Generously allowed regions, %	0.8

^1^ Gly 124- Tyr 135 were not restrained; ^2^ Based on residues Asn 3- Glu 123, excluding Gly.

### 3.5. Structure Description and Comparisons with other MA Structures

FIV MA is comprised of five alpha helices and two short beta sheet-like structures (Figure 4). Helices I, II, III, and V are packed against helix IV to form a globular protein. Residues Gly 2–Ser 6 appear to be unstructured based on the absence of amide-amide and amide-side chain NOEs (i to i+1 and i to i-1). Helix I spans from Arg 7 to Asn 18 and is positioned near the N terminus of helix II and the C terminus of helix IV. Despite the G6S mutation, there is no cap at the N terminus of helix I evidenced by the absence of NOEs from amide of Arg 7 to the alpha and beta protons of Ser 6. A cluster of positively charged residues comprised of Lys 26, Lys 28, and Lys 29 form a basic patch between helix I and II. Helix IV is the central helix of FIV MA, extending from Ser 77-Leu 96. Helix IV interacts with the other helices of the protein and is extremely hydrophobic, characteristic of the core of the protein. Helix V spans from Thr 102-Leu 115 and lays across helix IV. Helix V makes an interesting C-terminal cap between Tyr 110 and Leu 115. The aromatics of Tyr 110 are in close proximity with the beta, gamma protons of Leu 115, resulting in very upfield shifted Leu 115 side chain peaks. The side chains of residues Trp 9 (helix I), Phe 35 (helix II), and Phe 90 (helix IV) comprise a buried aromatic cluster. Residues following the C terminus of helix V are unstructured.

There are two adjacent beta interactions in the protein, both of which include residues in the flexible region near the C terminus of helix I. The first β interaction involves residues near the C terminus of helix I and the N terminus of helix II and include Val 19, Lys 28, Phe 30, and Val 21. The second β interaction involves residues near the C terminus of helix I and the N terminus of helix V and include Ala 20, Ser 101, and Thr 102. These interactions stabilize Val 19, Ala 20, and Val 21 to keep Lys 26, Lys 28, and Lys 29 (basic patch) solvent exposed. The position of the myristyl group was determined through the collection and analysis of the 2D ^1^H-^1^H NOESY and 2D ^1^H-^13^C HMQC NOESY (Figure 3). The terminal methyl group of the myristyl group (C^14^H_3_) packs in close proximity to the side chains of Trp 9, Phe 35 and Leu 60 while the methylenes (C^4−11^H_2_), which are degenerate and cannot be individually assigned, interact with the side chains of Trp 9, Ile 53 and Glu 55. The myristyl group penetrates about 10.6 Å from the surface of the protein.

**Figure 5:**
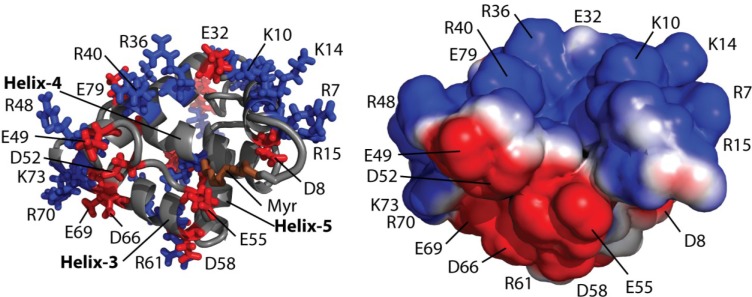
(Left) Cartoon representation of FIV myrMA^Q5A/G6S^ highlighting basic (blue) and acidic (red) residues as sticks. (Right) Electrostatic surface map showing a basic face (blue) and acidic face (red) of FIV myrMA^Q5A/G6S^.

FIV myrMA is a positively charged protein at physiologic conditions and creates a positively charged face, with a series of basic residues including Arg 7, Lys 10, Lys 14, Arg 15, Arg 36, Arg 40, and Arg 48. Interestingly, several negatively charged residues are distributed on the opposite face of the protein (Glu 49, Asp 52, Glu 55, Glu 69, Asp 66, and Asp 58), resulting in a bipolar protein surface (Figure 5). The positive surface resides near the myristate binding pocket and likely serves as the site of membrane binding.

The structure of FIV myr(-)MA was recently solved using X-ray crystallography [43]. The structure of FIV myrMA^Q5A/G6S^ is similar to that of FIV myr(-)MA. The backbone atoms of residues Asn 3- Glu 130 are well defined and are in good agreement with coordinates of FIV myr(-)MA. The linker between helices I and II are glycine rich and flexible. As such, this region of the protein is less well defined. We do not observe NOEs between the amide of Gly 22 to the side chain of Ser 27 or from the amide of Gly 24 to the side chain of Ser 27, which are in close proximity in the crystal structure. It is likely that the protein structure is compact in this region within the crystal and more dynamic in solution. Guillon and colleagues [43] also observe a short helix turn near the C- terminus of the protein. Our data show no secondary structure after helix V.

Serriere *et al.* [43] suggest two possible locations of the myristyl group using the molecular docking program AutoDock. The first putative myristyl position is close to Trp 9, Phe 35, Ile 39, Ile 53, and Phe 90, which is in agreement with our observations. The second proposed site is close to Ala 12, Arg 15, Glu 55, and Leu 95. We have no evidence of the myristyl group being in proximity to these residues.

Trp 9 alpha protons are in close proximity to Phe 90 aromatic protons in both the FIV myr(-)MA and myrMA structures. In the ^1^H-^1^H 2D NOESY of myrMA, however, Phe 90.hζ exhibits an upfield shifted resonance as compared to the ^1^H-^1^H 2D NOESY of myr(-)MA. It is plausible that as the myristyl group packs into the protein, there is a slight shift that causes Phe 90 and Trp 9 to come in closer proximity, resulting in this upfield shifted peak of Phe 90 aromatics. However, these rearrangements would be very small, as the NOEs associated with Phe90 are similar in the myrMA and myr(-)MA proteins.

**Figure 6:**
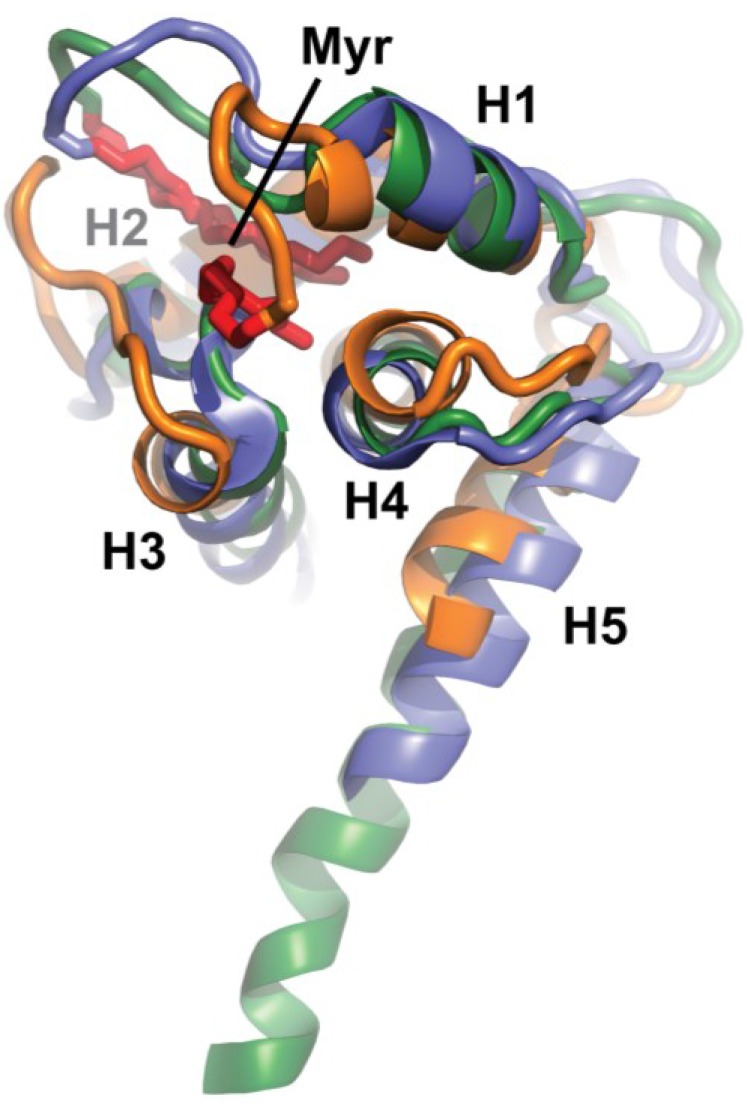
Best-fit superposition of helix backbone atoms of representative myrMA^Q5A/G6S^ (orange), myrHIV-1 MA (blue, PDB: 2H3I) and myrHIV-2 MA (green, PDB 2K4H), showing the high degree of similarity in structural architecture and location of the sequestered myristyl groups (red).

The global structure of FIV myrMA^Q5A/G6S^ is similar to the structures observed for the HIV-1 MA and HIV-2 MA proteins (Figure 6). Helices I, II, III, and V are similarly positioned around helix IV. Additionally, the myristyl group of both proteins lies in homologous regions. Notably, the myristyl group of FIV myrMA^Q5A/G6S^ packs in a hydrophobic pocket in a manner similar to that observed for the HIV-1 and -2 myrMA proteins, making contacts with the branched side chains of several buried hydrophobic residues, as well as with the aromatic side chains of Phe90 and Trp9. There are, however, minor differences between the structures. Helix I of HIV-1 MA and HIV-2 MA form an uncommon 3_10_ helix whereas helix I of FIV MA has a more frequently observed 4_13_ helix. Whereas there is no noted secondary structure between helix I and II of HIV-1 MA, there are two small beta interactions that keep Lys 26, Lys 28 and Lys 29 solvent exposed. The likely primary function of this basic patch is to make electrostatic interactions with the plasma membrane, although it has been suggested that the basic patch may also serve to interact with the viral genome [66]. FIV MA has a longer region between helix I and II and fewer positively charged residues than HIV-1 MA and HIV-2 MA. This may allow for more flexibility to interact with the PM. The C-terminal helices of FIV myrMA^Q5A/G6S^ and HIV-2 MA (helix V) NMR structures are shorter than the extended helix V of HIV-1 MA crystal structure, possibly due to trimer-dependent intermolecular packing. FIV myrMA^Q5A/G6S^ also bears close similarity to SIV MA (PDB: 1ECW) and EIAV MA (PDB: 1HEK), although EIAV is not N-terminally myristylated. Notably, FIV MA does not bear any similarity to the myristylated MoMuLV MA (PDB: 1MN8). Although MoMuLV MA has 5 alpha helices similar to most other retroviral MA proteins, the orientation of the helices is significantly different.

### 3.6. FIV Gag Assembly and Release in Cells are Modulated by PI(4,5)P_2_

Depletion of PI(4,5)P_2_ from the PM causes HIV-1 Gag relocalization to internal membranes and results in a decrease in viral release efficiency [14]. Similar observations have been made for HIV-2 [24]. In addition, expression of a constitutively active human Arf6/Q67L mutant in HeLa cells, which causes continuous synthesis of PI(4,5)P_2_, has been shown to sequester PI(4,5)P_2_ away from the PM into endosomal vesicles, which restricts HIV-1 release [14].

**Figure 7:**
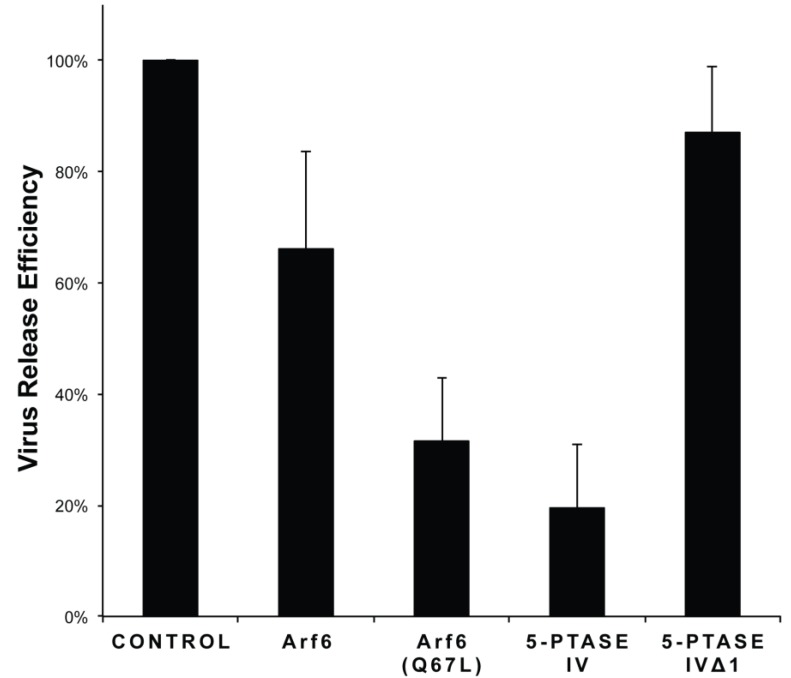
PI(4,5)P_2_ disruption impairs FIV particle production. Feline CRFK cells were co-transfected with pFIV-Orf2rep and plasmids expressing human Arf6 in either its native or constitutively active form (Q67L) or human 5-phosphatase-IV (5ptaseIV) in its native or catalytically inactive form (5ptase Δ1) using Lipofectamine LTX PLUS reagent (Invitrogen), at a DNA ratio of 9:1 (FIV:Arf6/5ptaseIV). Cells were metabolically labeled with [^35^S] Met/Cys and FIV proteins were immunoprecipitated from cell and viral lysates with anti-FIV p24gag (see Materials and Methods). Virus release efficiency was calculated as the proportion of virion-associated FIV capsid compared with total FIV Gag, normalized to samples containing FIV plasmid alone. Error bars indicate standard error of the mean (SEM) from at least 3 independent experiments. Reductions in virus release in the presence of Arf6/Q67L and 5ptaseIV were statistically significant (paired Student’s *t*-test).

To determine the effect of PI(4,5)P_2_ localization on FIV release, we co-transfected CRFK cells with the FIV molecular clone pFIV-Orf2rep and plasmids expressing either 5-phosphatase IV (to deplete PI(4,5)P_2_ from the PM), Arf6, or Arf6/Q67L (to induce PI(4,5)P_2_ relocalization in aberrant endosomal structures) [14]. FIV release efficiency was restricted in CRFK cells when PI(4,5)P_2_ levels were perturbed by expression of either human 5ptaseIV or human Arf6/Q67L (Figure 7). FIV release efficiency was markedly reduced in cells expressing 5-phosphatase IV (5ptase IV) as compared to the control. Similarly, cells expressing Arf6 or Arf6/Q67L have moderately reduced viral release. Notably, cells expressing the enzymatically inactive 5ptase IV derivative, 5ptase/∆1, [14] show undisrupted FIV release efficiency. These results suggest that the assembly and release of FIV particles from feline cells is highly sensitive to PM levels of PI(4,5)P_2_, similar to HIV-1 and HIV-2 [14,24].

Previous studies have shown that soluble PI(4,5)P_2_ analogs containing truncated acyl chains are capable of binding to a cleft on the surface of the HIV-1 myrMA protein and triggering myristate exposure [17]. However, we have recently found that these PI(4,5)P_2_ analogs also bind with similar affinities to a variety of non-membrane proteins (unpublished results). In fact, detergents with truncated acyl chains can function as general probes for hydrophobic patches on the surfaces of cytoplasmic proteins [67]. Although ^1^H-^15^N HSQC titration experiments indicate that PI(4,5)P_2_ analogs are capable of binding FIV myrMA (data not shown), we prefer to defer structural and mechanistic analyses until studies can be conducted with native PI(4,5)P_2_ using an appropriate membrane-like environment (studies are underway).

## 4. Discussion

Recent biophysical and X-ray structural studies of the unmyristylated form of the FIV MA protein provided a number of important insights [43]. Guillon and co-workers showed that the FIV myr(-)MA adopts a highly helical three dimensional structure that is similar to structures observed previously by NMR and X-ray crystallography for the HIV-1 and HIV-2 myr(-)MA and myrMA proteins [17,24,44,46,48,68,69]. Their studies showed that residues near the C-terminal end of the protein, which are disordered in the HIV MA NMR and X-ray structures, make intermolecular crystal contacts, and protein cross-linking studies suggested that the protein forms dimers under mild acidic conditions [43]. However, their studies also showed that deletion of a large C-terminal fragment of MA that included residues involved in intermolecular crystal contacts did not affect dimerization in solution, and quantitative analyses of the crystal structures with PISA [70] did not lead to identification of a functional dimer interface. In addition, docking studies identified two potential sites for myristate binding: one that is similar to the sequestration site observed for the HIV-1 and -2 myrMA proteins, and a second site that appears as a nearby hydrophobic groove on the surface of the protein. Thus, questions remained regarding the location of the myristate binding site, potential structural changes associated with myristylation, and the interface responsible for protein self-association in solution.

The present studies extend the work of Guillon and co-workers by focusing on a myristylated form of FIV MA. Our attempts to prepare the native, myristylated protein using a dual expression system encoding the yNMT enzyme were unsuccessful, due to the inability of yNMT to recognize and myristylate the wild-type protein. This interesting problem is discussed further below. We were, however, able to obtain significant myristylation levels for a mutant construct containing N-terminal residues that more closely match those of the HIV MA protein. Our rationale for this approach was that these residues are either unstructured, or appeared to be highly mobile, in the HIV-1 and HIV-2 myrMA proteins, and as such, we speculated that mutations at these sites would be tolerated. Indeed, transfection and mutagenesis studies demonstrate that these substitutions do not affect virus assembly or release from a feline kidney cell line. No concentration-dependent NMR chemical shifts were observed, indicating that the myristyl group, and the protein in general, adopts a unique monomeric conformation under the conditions employed (pH 7.0). This suggests that the FIV MA myristyl switch may be regulated by phosphoinositide binding. Our studies further indicate that the myristyl group is sequestered within a hydrophobic cavity in a manner similar to that observed for the HIV-1 and HIV-2 myrMA proteins [24,69]. The NMR signals of the aromatic protons of Phe 90 are shifted slightly relative to signals observed for the unmyristylated protein, and this is indicative of a structural difference. However, chemical shifts of protons near aromatic groups can be highly sensitive to very small structural perturbations, and since the NOE patterns of the myrMA and myr(-)MA proteins are highly similar, the structural perturbation that accommodates myristate sequestration must be very small (within the limits of measurement by the NOE experiments employed). We did not observe evidence for myristate binding to the alternative hydrophobic cleft site identified by computational docking [43].

The C-terminal tail of FIV myrMA contains three positively charged residues and three negatively charged residues that could potentially interact with the charged surface of the folded portion of the protein. However, our studies indicate that the tail is disordered, and no NMR evidence for intra- or intermolecular contacts was obtained.

An additional goal of our studies was to determine if FIV utilizes the cellular phosphoinositide signaling system as a means for targeting Gag proteins to the PM for virus assembly. Virus assembly studies demonstrate that FIV release efficiency is severely restricted from cells with either attenuated PI(4,5)P_2_ levels in the PM, or enhanced PI(4,5)P_2_ levels in endosomal membranes. Although solubility issues precluded quantitative PI(4,5)P_2_ binding studies, our *in vitro* titration experiments demonstrate that FIV myrMA is capable of interacting with PI(4,5)P_2_. Collectively, these results provide evidence that, as observed for HIV-1 and HIV-2, FIV utilizes the PI(4,5)P_2_ signaling system for late phase intracellular Gag trafficking and membrane targeting.

An interesting and unanswered question that has emerged from these studies is: Why does FIV employ a non-consensus MA myristylation signal? Whereas the prototypical mammalian NMT recognition signal is M-G-X-X-X-S/T (X = variable amino acid), the FIV MA myristylation signal is M-G-X-X-X-G. One possible explanation is that felines generally encode the M-G-X-X-X-G myristylation signal, and FIV has adapted to its host species. To investigate this possibility, we analyzed a variety of proteins that are myristylated in humans, and identified the homologous proteins in felines [71]. In nearly all cases, the myristyl signals employed by the cellular proteins of felines matched those of the corresponding human proteins (M-G-X-X-X-S/T; Supplementary Table S1). A second possible explanation is that the specific isolate of FIV used in our studies is divergent from other FIV isolates. However, analysis of the amino acid sequences of the 25 reported isolates of FIV revealed that, in all cases, the FIV MA myristylation signal includes a glycine in the 6th position (M-G-X-X-X-G).

The above analysis shows that most myristylated feline host proteins contain a prototypical mammalian myristylation signal (M-G-X-X-X-S/T). In rare cases, the less conserved myristyl signal is utilized. For example, the feline protein ADP ribosylation factor 4 is a myristylated protein that has a non-consensus glycine in the 6th position. The human form of the protein also has a glycine in the 6th position, supporting its use as a myristyl signal, albeit rarely used. More interesting, however, is that this less common myristyl signal (M-G-X-X-X-G) is conserved across the 25 known isolates of FIV. Other retroviruses, including HIV-1, HIV-2, SIV and bovine leukemia virus, also utilize the more common mammalian myristylation motif. It appears that FIV has evolved to prefer glycine instead of Ser/Thr in the 6th position, making the yNMT enzyme used in our expression system unable to myristylate wild-type FIV MA protein.

It remains unclear to us as to why FIV MA utilizes a non-consensus myristylation signal, even though most feline host proteins employ the more common mammalian myristylation signal. Considering that all of the FIV isolates we examined have this uncommon signal, there must be an evolutionary advantage for its use. The non-prototypical signal is used in human and feline host proteins, but only rarely. It is possible that because yeast NMT (non-permissive to G6) is less evolved than feline NMT (G6 permissive), Gly 6 represents an alternative, more evolved myristylation signal. Alternately, there may be an unidentified structural or functional advantage of using Gly 6 in the function of FIV MA within the host cell. Our initial hypothesis that a serine at position 6 would adopt a commonly observed N-cap structure, and that this might be structurally and/or evolutionarily disadvantageous, was not supported by our structural findings. Comparative structural and biophysical studies involving the myristylated wild-type protein could shed light on this unresolved question.

## Supplementary Files

Supplementary File 1
