# Supplementary material for: NMR Structure of the Myristylated Feline Immunodeficiency Virus Matrix Protein
Source: Viruses. 2015 Apr 30;7(5):2210–29. doi: 10.3390/v7052210 (PMC4452903; doi:10.3390/v7052210)
Supplement: Supplementary File 1 [file viruses-07-02210-s001.pdf]

Supplementary Materials

NMR Structure of the Myristylated Feline Immunodeficiency Virus Matrix Protein

Lola A. Brown [1], Cassiah Cox [1], Janae Baptiste [1], Holly Summers [1], Ryan Button [1], Kennedy Bahlow [1], Vaughn Spurrier [1], Jenna Kyser [1], Benjamin G. Luttge [2], Lillian Kuo [2], Eric O. Freed [2,*] and Michael F. Summers [1,*]

[1] Howard Hughes Medical Institute, University of Maryland Baltimore County, 1000 Hilltop Circle, Baltimore, MD 21250, USA; E-Mails: lola.brown@yale.edu (L.A.B.); cassiah.cox@nih.gov (C.C.); janaeb@umbc.edu (J.B.); hsummers@umbc.edu (H.S.); ryan.button@umaryland.edu (R.B.); kennedy.bahlow@temple.edu (K.B.); vspurrier@uchicago.edu (V.S.); jmk216@lehigh.edu (J.K.); summers@hhmi.umbc.edu (M.F.S.)

[2] Virus-Cell Interaction Section, HIV Drug Resistance Program, National Cancer Institute at Frederick, Frederick, MD 21702-1201, USA; E-Mails: bxl244@case.edu (B.G.L.); kuols@mail.nih.gov (L.K.)

* Authors to whom correspondence should be addressed; E-Mails: efreed@mail.nih.gov (E.O.F.); summers@hhmi.umbc.edu (M.F.S.); Tel.: +1-301-846-6223 (E.O.F.); +1-410-455-2527 (M.F.S).

Table S1. Myristylation signal of myristylated feline proteins. Note that many myristylated feline proteins have the consensus mammalian myristyl signal M-G-X-X-X-S pattern. The sixth residue is in the bolded box.

Feline Protein	Myristyl Signal						Accession Number
Recoverin	**M**	**G**	N	S	K	**S**	XP_003996258.1
neuronal tissue-enriched acidic protein	**M**	**G**	G	K	L	**S**	XP_003981588
tyrosine-protein kinase Lyn isoform 1	**M**	**G**	C	I	K	**S**	XP_003999866.1
Tyrosine-protein kinase Yes	**M**	**G**	C	I	K	**S**	XP_003995101.1
nitric oxide synthase	**M**	**G**	N	L	K	**S**	XP_003983267.1
calcineurin subunit B type 2	**M**	**G**	N	E	A	**S**	XP_003995756.1
calcinuerin type 2	**M**	**G**	N	E	A	**S**	XP_003995756.1
ADP-ribosylation factor 4	**M**	**G**	L	T	I	**S**	XP_003982414.1
Paladin	**M**	**G**	T	T	A	**S**	XP_003994073
tyrosine-protein kinase –SRC	**M**	**G**	S	N	K	**S**	XP_003994073
NADH cytochrome reductase	**M**	**G**	F	Q	P	**S**	XP_003999428.1
ADP-ribosylation factor 3	**M**	**G**	N	I	F	G	XP_003988679.1
annexin XIII	**M**	**G**	N	R	H	A	XP_004000148.1
myristilated and palmitylated serine-threonine kinase MPSK	**M**	**G**	H	A	L	C	XP_003991222.1
tyrosine-protein kinase Fgr	**M**	**G**	C	V	F	C	XP_003989804.1

Table S2. Sequence alignment of first twelve amino acid residues of several isolates of FIV. Glycine 6 (bolded column) is conserved throughout the species (FIV sequences kindly provided by Brian Foley, HIV databases, www.hiv.lanl.gov).

FIV Isolate	Myristyl Signal											
FIVPETLM	**M**	**G**	N	G	Q	**G**	R	D	W	K	M	A
FIVZ1 | X57002	**M**	**G**	N	G	Q	**G**	R	D	W	K	M	A
FIVUSIL2489	**M**	**G**	N	G	Q	**G**	R	D	W	K	M	A
FIV-C | AF474246	**M**	**G**	N	G	Q	**G**	R	D	W	K	V	A
FIVUSCAPPR	**M**	**G**	N	G	Q	**G**	R	D	W	K	M	A
FIVJPTM2	**M**	**G**	N	G	Q	**G**	R	D	W	K	M	A
FIVPleE | EU117992	**M**	**G**	N	E	Q	**G**	K	E	V	K	A	A
FIVKZOMA	**M**	**G**	N	E	Q	**G**	K	E	V	K	A	A
FIVPleB | EU117991	**M**	**G**	N	E	Q	**G**	K	E	V	K	A	A
PLB14CG	**M**	**G**	N	N	Q	**G**	K	E	L	K	A	A
PLV_vanc | DQ192583	**M**	**G**	N	E	S	**G**	K	E	E	R	I	I
PCOJM01 | EF455609	**M**	**G**	N	E	S	**G**	K	E	E	R	V	I
PCOYM137 | EF455609	**M**	**G**	N	E	S	**G**	K	E	E	R	V	I
PCOYM137 | EF455609	**M**	**G**	N	E	S	**G**	K	E	E	R	V	I
PCOYM137 | EF455611	**M**	**G**	N	E	S	**G**	K	E	E	R	V	I
PCOJF6 | EF455610	**M**	**G**	N	E	S	**G**	K	E	E	R	V	I
PCOSR631 | EF455613	**M**	**G**	N	E	S	**G**	K	E	E	R	V	I
PCOSR631 | EF455614	**M**	**G**	N	E	S	**G**	K	E	E	R	V	I
PCOYF127 | EF455612	**M**	**G**	N	E	S	**G**	K	E	E	R	V	I
PCOGC34 | EF455603	**M**	**G**	N	E	S	**G**	K	E	E	R	V	I
PCOMC350 | EF455604	**M**	**G**	N	E	S	**G**	K	E	E	R	V	I
PCOMC121 | EF455606	**M**	**G**	N	E	S	**G**	K	E	E	R	V	I
PCOYM29 | EF455607	**M**	**G**	N	E	S	**G**	K	E	E	R	V	I
PCOYF16 | EF455608	**M**	**G**	N	E	S	**G**	K	E	E	R	V	I
PCOCoLV |EF455615	**M**	**G**	N	E	S	**G**	K	E	E	R	V	I
PCOMC100 | EF455605	**M**	**G**	N	E	S	**G**	K	E	E	R	I	I
